# Cellular T-cell immune response profiling by tetravalent dengue subunit vaccine (DSV4) candidate in mice

**DOI:** 10.3389/fimmu.2023.1128784

**Published:** 2023-02-28

**Authors:** Charu Aggarwal, Viswanathan Ramasamy, Amit Garg, Rahul Shukla, Navin Khanna

**Affiliations:** ^1^ Translational Health, Molecular Medicine Division, International Centre for Genetic Engineering and Biotechnology, New Delhi, India; ^2^ Division of Virus Research and Therapeutics, CSIR-Central Drug Research Institute, Lucknow, India; ^3^ Translational Health Science and Technology Institute, NCR Biotech Science Cluster, Faridabad, India

**Keywords:** dengue vaccine, DSV4, BALB/c, type-specific immunity, ADE, T-cell, TNF-α, IFN-γ

## Abstract

While most vaccines aim to develop a solid humoral and neutralizing antibody response against the pathogen, an effective vaccine candidate should be able to stimulate both the B-cell mediated humoral immunity, and T-cell mediated cellular immunity. The focus of vaccinology is rapidly gaining to generate T cell responses, which can mediate pathogen clearance and help B cells leading to protective antibody responses. Here we evaluate the cellular immune response of the pre-clinical tetravalent dengue subunit vaccine candidate, DSV4, in mice. While we have shown previously that DSV4 induces type-specific neutralizing antibody responses in mice, in this study, we show that the vaccine candidate DSV4 well induces dengue-specific T- cell responses evaluated by their ability to produce IFN-γ. In addition to IFN-γ secretion by both CD4+ and CD8+ T-cells in immunized mice, we observed that DSV4 also induces a higher frequency and cytokine functions of follicular CD4+ helper T-cells (T_FH_). These cytokines lead to an efficient germinal center reaction and potent B cell antibody response. Apart from T_FH_ response, DSV4 stimulated Type 1 T helper cells (T_H1_) which is characteristic of a viral infection leading to secretion of pro-inflammatory cytokines and phagocyte-dependent protective immune responses. Our study highlights that DSV4 can mediate both arms of adaptive immunity-humoral and cell-mediated immunity in mice. By elucidating vaccine-specific T cell response, our work has implications in showing DSV4 as an effective, type-specific and safe dengue vaccine candidate.

## Introduction

Dengue is a significant rising public health threat globally, resulting in millions of cases and deaths annually (1, 2). The spread of dengue infection has increased dramatically over the last few years in terms of geography and the number of incidences (3). Dengue virus affects people of all ages and causes many symptoms. Dengue-infected subjects show various signs, including asymptomatic infection to acute disease symptoms ranging from mild fever to severe conditions (4). The need for the dengue vaccine is of utmost importance as there are no approved anti-viral or therapeutics. With the increasing number of cases, dengue infection poses a substantial economic burden on the global healthcare system (5). The existence of four serotypes, namely DENV-1, -2, -3 and 4, pose complexity in developing a safe and effective dengue vaccine (6). These four serotypes are closely related and share almost 65% genome homology (7). However, infection with one serotype does not offer protection from the other three serotypes (8). Moreover, reinfection with a heterologous dengue virus serotype leads to a complicated and severe disease due to a phenomenon known as Antibody-Dependent Enhancement (ADE) (9, 10). Thus, an ideal dengue vaccine should elicit type-specific virus neutralization of all serotypes without resulting in ADE in humans.

Currently, only three dengue vaccines are either approved or at advanced stages of clinical trials (11). A vaccine named Dengvaxia, commercially produced by Sanofi, has been approved and introduced in many countries. This vaccine is a chimeric live attenuated tetravalent dengue vaccine consisting of all four-dengue serotype structural proteins on a yellow fever background. However, the vaccine is partially effective and approved for the 9-45 age group with prior exposure to dengue virus infection. Dengvaxia is contraindicated for dengue-naive individuals (12, 13). Recently, Indonesia approved Takeda’s live-attenuated dengue vaccine (TAK003) for both dengue-naive and exposed individuals. The NIAID dengue vaccine (TV003) comprises live attenuated forms of all four dengue virus serotypes and is currently in Phase III human trials (14). These vaccines can generate dengue-neutralizing antibodies and dengue-specific T-cell response. In a 3-year follow-up post-vaccination, the Takeda vaccine has exhibited the potential to enhance disease for seronegative individuals infected with the dengue virus-3 serotype (15). However, no information is available on the ADE potential of the NIAID dengue candidate vaccine.

More recently, our group have designed a recombinant tetravalent dengue subunit vaccine candidate, DSV4, which is based on the self-assembling nature of Hepatitis B virus (HBV) surface antigen, fused tandemly with envelope domain III (EDIII) region of all four dengue virus serotypes (DENV-1: West Pac-74, DENV-2 PR159-S1/69, DENV-3: H87/56 and DENV-4 H241-P) and expressed in yeast, *Pichia pastoris* as a chimeric Virus Like Particles (VLP) (16). The type-specific neutralizing EDIII antibodies are the most potent blockers of virus entry and are devoid of ADE potential (17). Thus, DSV4 elicited immune responses minimize the risk of severe dengue. Indeed, the ability of DSV4 vaccine candidate to predominantly stimulate dengue type-specific neutralizing antibodies without the risk of ADE is well-documented in mice and macaques (16).

Further, it is becoming increasingly evident that a successful and effective vaccine candidate for any infection needs to activate both humoral neutralizing antibody response and cellular T cell immunity. T cells are the significant players in the cell-mediated immune response. They help eliminate infection by its cytolytic activities and provide critical help for generating high-affinity B cells and memory responses. Thus, an ideal, practical and safe dengue vaccine would be one that not only generates serotype-specific neutralizing antibodies, circumventing ADE but also results in the induction of T cell responses against the dengue virus. Previous studies have shown that the primary T cell epitopes against dengue virus are non-structural proteins, namely NS1, NS3 and NS5 (18, 19). Although antibodies targeting EDIII are type-specific, it is unclear if EDIII-based DSV4 elicits a protective T-cell response.

Here, in this study, we have looked at the ability of DSV4, an EDIII-based dengue subunit vaccine (16), to elicit a T-cell response in immunized mice. We observed that DSV4 immunization in mice results in the induction of dengue EDIII-specific IFN-γ+ T cells. Additionally, vaccinating mice with DSV4 also leads to the generation of T_FH_ effector CD4+ T cells and polyfunctional T_H1_ CD4+ T cells. These observations suggest DSV4 is a safe, effective vaccine candidate by its ability to generate T cell response and the type-specific humoral response. Further detailed analysis of the induced T cell response is needed to define the true potential of DSV4 as an effective dengue vaccine candidate.

## Methods

### Ethics statement

All the experiments using BALB/c mice were conducted at International Centre for Genetic Engineering and Biotechnology (ICGEB), New Delhi (IAEC No.: ICGEB/IAEC/07032020), in accordance with Experimental Animals (CPCSEA) guidelines of Government of India.

### Immunization of mice with DSV4 and collection of spleen

Four-six week old BALB/c mice (n=5) was immunized intramuscularly (i.m) with 20 μg of DSV4 adsorbed on 500 μg alhydrogel on days 0, 21 and 42. Blood was collected on day 56 for evaluation of antibody titers by ELISA and FACS-based virus-neutralization assay against all four dengue serotypes. Furthermore, the mice were euthanized on day 56 post the final boost, and splenocytes were isolated. Briefly, the spleen was mashed on the 70 μm cell strainer using a plunger from a 5 ml syringe, and the cell suspension was washed with 1% RPMI [1% (v/v) Δ FBS + 1x RPMI] at 1500 rpm for 5 mins. The cell pellet was lysed with RBC lysis buffer for 2-3 mins and then again washed with 1% RPMI. Thereafter, the cell pellet was resuspended in 10% RPMI [10% (v/v) Δ FBS + 1x RPMI], and cells were counted using haemocytometer.

### Ex-Vivo T-cell peptide stimulation

The freshly isolated splenocytes (2x10^6^ cells/well) were stimulated for 6 -hours with or without stimulant. For antigen-specific stimulation, dengue EDIII peptide pool of dengue serotypes 1, 2, 3 and 4 and Hepatitis B surface antigen peptides (Custom synthesized from GenScript, USA; **Supplementary Tables 1, 2**) were used. These EDIII peptides were reconstituted with dimethyl sulfoxide (DMSO) and were combined to make a dengue EDIII 1-4 peptide pool, as summarized in **Supplementary Table 1**. The final concentration of individual peptides at the stimulation time is 10 μg/ml. As a positive control, the cells were stimulated with PMA/I at 1X concentration (cell stimulation cocktail; eBioscience, Cat# 00-4970-03). After 2- hours of stimulation at 37 °C, brefeldin A was added at 1X concentration (Protein Transport Inhibitor Cocktail, eBioscience, Cat#00-4980-93). The cells were cultured for 4- hours at 37 °C, with 5% CO_2_. The cells were harvested and stained for flow cytometry.

### Intracellular flow cytometry analysis

The stimulated splenocytes were harvested at the end of 6- hours and washed with ice-cold FACS buffer (0.25% FBS in 1X PBS). Cells were blocked with 5 μg/ml Fc Block (anti-mouse CD16/CD32; clone 2.4G2) for 20 mins on ice and then surface stained for 30 mins on ice with CD3 (145-2C11), CD4 (GK 1.5), CD8 (53-6.7), CD69 (H1.2F3), CXCR5 (2G8), ICOS (15F9), PD-1 (29F.1A12), and CXCR3 (CXCR3-173). Cells were then washed with FACS buffer and fixed with FOXP3/Transcription Factor staining buffer set (eBioscience, Cat# 00-5523-00) for 45-60 mins on ice. The cells were washed with Perm Buffer followed by intracellular staining for 60 mins with Ki67 (11F6), IFN-γ (XMG1.2), TNF-α (MP6-XT22), IL-2(JES6-5H4), IL-21(FFA21), IL-10 (JES5-16E3), T-Bet (4B10), GATA3 (L50-823) and BCL-6(K112-91), diluted in perm buffer. The fixable viability dye eFluor 780 (eBioscience, Cat# 65-0865-18) to exclude dead cells. The cells were washed and acquired on LSR-II (BD) and analyzed using FlowJo software (TreeStar Inc.).

### IFN-γ enzyme-linked immunosorbent spot (ELISpot) assay

Freshly isolated splenocytes (1x10^6^ cells) were added onto PVDF plates (Millipore, Cat# MSIPS4510) coated 10 μg/ml IFN-γ (MABTECH Cat# 3321-3-1000). Cells were either unstimulated or stimulated with dengue EDIII 1-4 peptide pool (10 μg/ml; **Supplementary Table 1**), HBsAg peptide pool (10 μg/ml; **Supplementary Table 2**) and PMA/I (1X) for 16-18 hours. The washed plate was incubated with IFN-γ biotin (MABTECH Cat# 3321-6-1000), washed again, incubated with streptavidin-HRP (MABTECH Cat# 3310-9-1000), and detected using AEC substrate. Plates were imaged using ImmunoSpot Analyzer and spots were counted.

## Results

### DSV4 immunization in mice induces IFN-γ secretion by T-cells

Cellular immunity is instrumental in preventing an infection. The tetravalent dengue subunit vaccine candidate, DSV4 induced a robust type-specific neutralizing antibody response in mice and also did not promote ADE in AG129 mouse model (16). Further, to understand the cellular immune response induced by the vaccine candidate DSV4, we looked at the dengue-specific IFN-γ response by T cells. IFN-γ-secreting T-cell frequencies are widely taken as an effective parameter to test the vaccine-induced cellular immune response. We evaluated the IFN-γ T cell response by intracellular flow cytometric staining and IFN-γ ELISpot assay.

**Figure 1:**
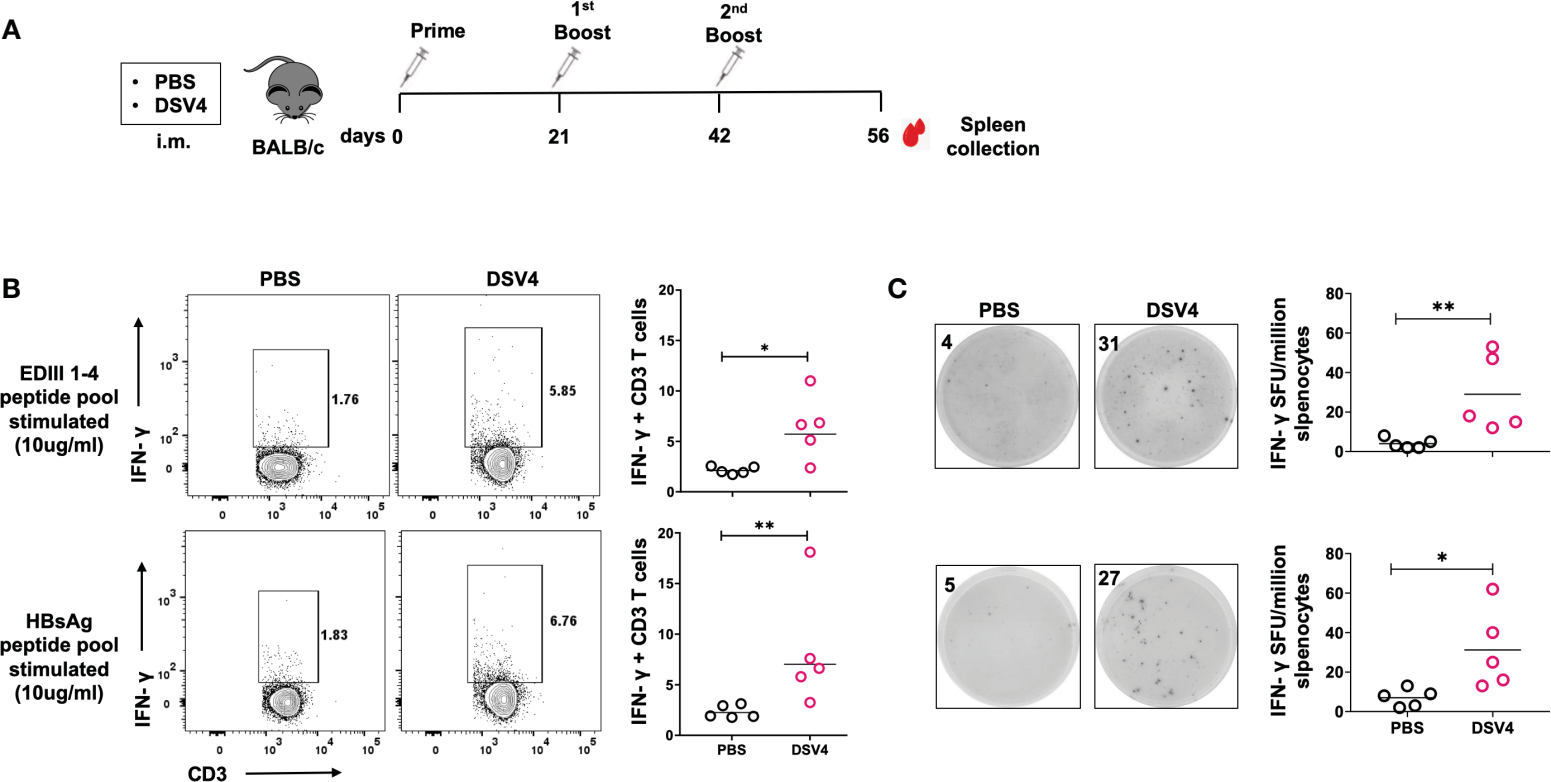
IFN-γ response by CD3+ T cells in DSV4 immunized mice. **(A)** Immunization schedule of DSV4 in BALB/c mice. Four to six weeks old BALB/c (n=5) mice were immunized either with 1X PBS (mixed with 500 μg of alum) or adjuvanted vaccine candidate, DSV4 (20 μg) following day 0, 21 and 42 immunization schedule shown in the schematic diagram. Mice were sacrificed after 14 days of final boost and their spleen was harvested and splenocytes were isolated. **(B)** Flow cytometric analysis of IFN-γ secretion by CD3+ T cells were evaluated where splenocytes were isolated from mice and analyzed for intracellular IFN-γ secretion by T cells by flow cytometry in mice immunized with DSV4. Mice immunized with PBS were taken as control. Top panel and bottom panel show the representative contour plots for the IFN-γ secretion by T cells in splenocytes stimulated with EDIII1-4 peptide pool (10 μg/ml) and HBsAg peptide pool (10 μg/ml) respectively for 6- hours. The numbers in the contour plot represents the frequency of the population within the gate. Scatter plot represents the IFN-γ response in all the 5 mice within the experiment. **(C)** IFN-γ ELISpot of DSV4 immunized mice. The representative ELISpot pictures showing IFN-γ secretion by splenocytes of mice stimulated with EDIII1-4 peptide pool (10 μg/ml; top panel) and HBsAg peptide pool (10 μg/ml; bottom panel) for 16-18 hours. The numbers in the box represents the spot forming unit (SFU)/number of spots within a well. Scatter plot represents the IFN-γ response in all the 5 mice within the experiment. The line in the scatter plot represents the geometric mean of the IFN-γ response in all the 5 mice. Each data point in the scatter plot represents an individual animal. The data shown is of a single experiment representative of two independent experiments with four to five mice per group per experiment. *-p=0.01-0.05, **-p=0.001-0.01.

Four-six weeks old BALB/c mice (n=5) were immunized with either PBS control or dengue vaccine candidate, DSV4 (20 μg adsorbed on 500 μg of aluminium hydroxide) as shown in **Figure 1A**, and two weeks after the final DSV4 booster (2^nd^ booster) mice were euthanized, their spleen were collected, and splenocytes were isolated. The splenocytes from PBS control and DSV4 immunized mice were stimulated with the dengue EDIII 1-4 peptide pool (10 μg/ml) and Hepatitis surface B antigen (HBsAg) peptide pool (10 μg/ml). Following six hours of stimulation with the peptide pool, we observed significantly higher frequencies of IFN-γ-secreting T-cells in DSV4 immunized mice compared to PBS control mice (**Figure 1B**) by intracellular flow cytometry staining. A considerably higher IFN-γ SFU/million splenocytes were detected by IFN-γ T cell ELISpot assay in both dengue EDIII 1-4 peptide pool stimulated and HBsAg peptide pool stimulated among DSV4 immunized mice (**Figure 1C**). In contrast, PBS control mice had negligible spots. Thus, the dengue vaccine candidate DSV4, a virus-like particle (VLP) having EDIII domains of all four-dengue virus and Hepatitis B virus surface antigen; induced both dengue EDIII specific and HBsAg specific cellular immune response. Since this study aims to understand vaccine-induced dengue-specific T cell response, we have focused on the immune response generated against dengue EDIII 1-4.

**Figure 2:**
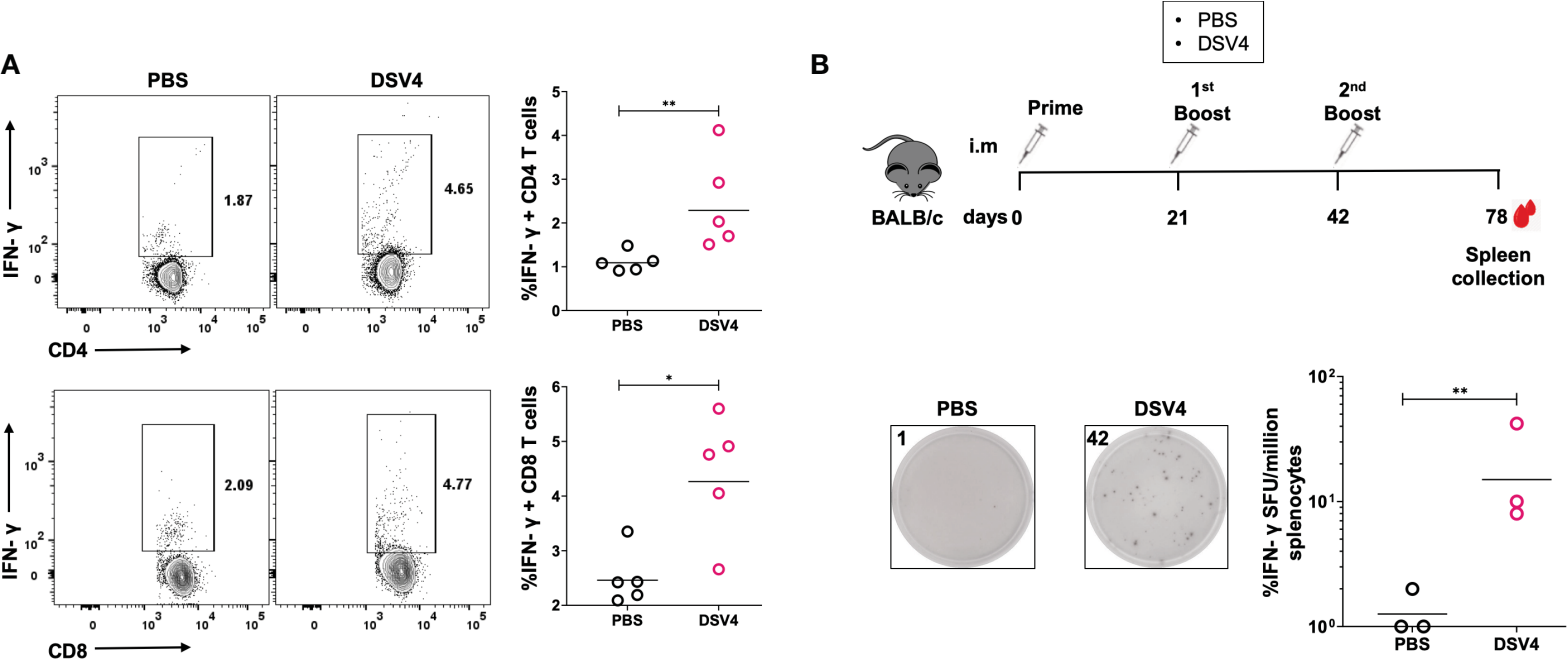
IFN-γ response by T cell subsets in DSV4 immunized mice. **(A)** Flow cytometric analysis of IFN-γ secretion by CD4+ and CD8+ T cell subsets. The isolated splenocytes were analyzed for intracellular IFN-γ secretion by T cells by flow cytometry in mice immunized with DSV4. The PBS plus alum injected mice were taken as control. Top panel and bottom panel show the representative contour plots for the IFN-γ secretion by CD4+ T cells (top panel) and CD8+ T cells (bottom panel) in splenocytes stimulated with EDIII1-4 peptide pool (10 μg/ml) for 6- hours. The numbers in the contour plot represents the frequency of the population within the gate. Scatter plot represents the IFN-γ response in all the 5 mice within the experiment. The line between the scatter plot represents the geometric mean of the IFN-γ response in all the 5 mice and each data points in the scatter plot represents an individual animal. The data shown here is of a single experiment representative of two independent experiments with n=4-5 mice per group per experiment. **(B)** Long term IFN-γ ELISpot of DSV4 immunized mice. Schematic (top) showing the immunization schedule. The mice were sacrificed after 36 days post final booster and their spleen isolated. Representative ELISpot pictures (bottom) showing IFN-γ secretion by splenocytes of mice stimulated with EDIII1-4 peptide pool (10 μg/ml) and with IL-2 (0.5 ng/ml, after every 48- hours) for 10 days; and were re-stimulated on 10^th^ day with EDIII1-4 peptide pool (10 μg/ml) for 16-18 hours. The numbers in the box represents the spot forming unit (SFU)/number of spots within a well. Scatter plot represents the IFN-γ response in all the 3 mice within the experiment. The geometric mean of the IFN-γ response in all 3 mice is represented by a line. *-p=0.01-0.05, **-p=0.001-0.01,.

Further, we evaluated the IFN-γ response in CD4+ and CD8+ T cells (**Figure 2A**) by intracellular flow cytometry IFN-γ staining. Both CD4+ and CD8+ T cells were stimulated with dengue EDIII 1-4 peptide pool secreted higher amounts of IFN-γ in DSV4 immunized mice compared to PBS control mice.

An active T cell response is sustained for 7-14 days after antigen stimulation, after which most of the antigen-specific T cells start dying while leaving behind a tiny proportion of antigen-specific memory T cells. These memory T cells are responsible for the recall responses during an infection after initial priming with the vaccine. To understand if our dengue vaccine candidate, DSV4, also induces T cell memory, we did a long-term antigen stimulation of the immunized mice with dengue EDIII 1-4 peptide pool along with IL-2 for ten days. We determined the memory T cell activation into effector IFN-γ-secreting T cells by ELISpot. The mice were immunized and euthanized, as shown in **Figure 2B** (top). The spleen was collected 36 days after the final boost to capture the antigen-specific memory T cells. The splenocytes were stimulated with EDIII 1-4 peptide pool (10 μg/ml) for ten days, and IL-2 (0.5 ng/ml) was added after every 48 hours. We observed a higher proportion of IFN-γ-secreting cells/million splenocytes in DSV4 immunized mice compared to PBS control mice (**Figure 2B**, bottom), showing that DSV4 can also induce long-term memory T cells post-immunization. Hence, our data indicates that DSV4 induces antigen-specific robust and long-lasting T cell responses in immunized mice.

### DSV4 immunization induces higher T_FH_ CD4+ T cells and its functions in BALB/c

**Figure 3:**
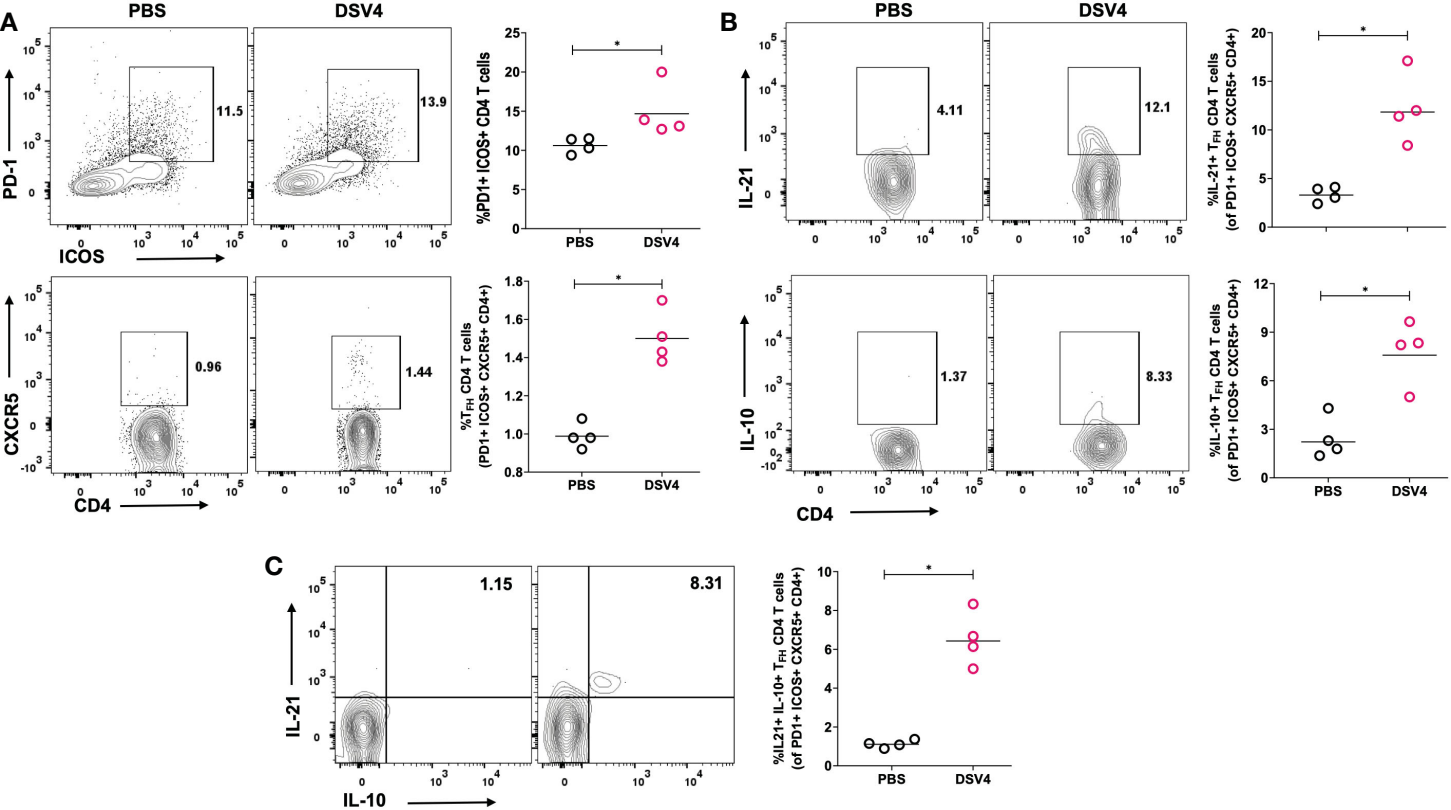
T_FH_ CD4+ T cell frequency and function in DSV4 immunized mice. **(A)** T_FH_ CD4+ T cell subset frequency in DSV4 immunized mice. Contour plots shows T_FH_ CD4+ T cell subset frequencies which were identified as PD1+ICOS+CXCR5+ within CD4+CD3+ gated cells by flow cytometry in mice immunized with DSV4 and stimulated with EDIII1-4 peptide pool (10 μg/ml) for 6- hours. PBS plus alum injected mice were taken as control. The numbers in the contour plot represents the frequency of the population within the gate. Scatter plot represents the PD1+ICOS+ CD4+ T cells gated on CD4+ CD3+ T cells (top panel) and CXCR5+ cells gated on PD1+ICOS+ CD4+ T cells (bottom panel) in all the 5 mice within the experiment. **(B)** Cytokine function of T_FH_ CD4+ T cell subset. Contour plots shows IL-21 secretion (top panel) and IL-10 (bottom panel) secretion by T_FH_ CD4+ T cell subsets by flow cytometry in mice immunized with DSV4 and stimulated with EDIII1-4 peptide pool (10 μg/ml) for 6- hours. Alum mixed PBS injected mice were taken as control. The numbers in the contour plot represents the frequency of the population within the gate. Scatter plot represents the IL-21+ (top) and IL-10+ (bottom) T_FH_ CD4+ T cell subsets gated on PD1+ICOS+CXCR5+ CD4+ T cells gated in all the 5 mice within the experiment. **(C)** IL 21- IL 10 co-producers. Contour plots shows IL 21 – IL 10 cp-producing T_FH_ CD4+ T cell subsets by flow cytometry in mice immunized with DSV4 and stimulated with EDIII1-4 peptide pool (10 μg/ml) for 6- hours. Mice immunized with PBS plus alum were taken as control. Scatter plot represents the IL-21+-IL10+ T_FH_ CD4+ T cell subsets gated on PD1+ICOS+CXCR5+ CD4+ T cells gated in all the 5 mice within the experiment. The line in the scatter plot represents the geometric mean of the IFN-γ response in all the 5 mice and each data point in the scatter plot represents an individual animal. The data shown is of a single experiment representative of two independent experiments with four to five mice per group per experiment. *-p=0.01-0.05.

The generation of potent antibody response requires an effective germinal center where B cells and follicular helper T cells (T_FH_) interact. T_FH_ are a specialized CD4+ T cell subset that resides in secondary lymphoid organs. They are critical in providing help to B cells and secrete cytokines that promote B cell survival and development of high affinity and long-lasting humoral antibody response. In this study, we have looked at the induction of T_FH_ CD4+ T cell response upon immunization with DSV4 vaccine candidate in mice. We observed that upon dengue EDIII 1-4 peptide pool stimulation, DSV4 immunization resulted in significantly higher T_FH_ CD4+ T cells identified within CD4+ gated cells as PD1+ICOS+CXCR5+ T-cells (**Figure 3A**). The T_FH_ CD4+ T cells provide the B cell help *via* the secretion of cytokines like IL-21 and IL-10 (**Figure 3B**) that allow survival and proliferation of affinity matured antigen-specific B cells. On evaluating the cytokine function of the T_FH_ CD4+ T cells in DSV4 immunized mice, we found that dengue EDIII 1-4 peptide pool stimulation resulted in a significantly higher proportion of IL-21+ and IL-10+ T_FH_ CD4+ T cells (**Figure 3C**). We also found a higher proportion of IL21+ IL10+ co-producing T_FH_ CD4+ T cells (**Figure 3C**, bottom) in mice after immunization with DSV4. In summary, our data show that immunization of mice with DSV4 resulted in activation and increase in T_FH_ CD4+ T-cells, which can secrete IL-21 and IL-10.

### DSV4 immunization elicits a TH1-dominated cellular immune profile in mice

**Figure 4:**
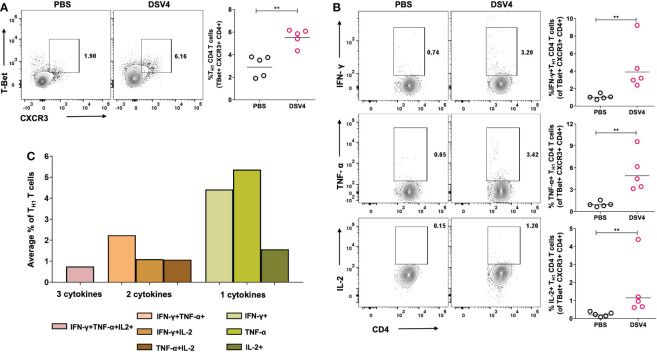
T_H1_ CD4+ T cell frequency and function in DSV4 immunized mice. **(A)** T_H1_ CD4+ T cell subset frequency in DSV4 immunized mice. Contour plots shows T_H1_ CD4+ T cell subset frequencies which were identified as T-Bet+CXCR3+ within CD4+CD3+ gated cells by flow cytometry in mice immunized with DSV4 and stimulated with EDIII1-4 peptide pool (10 μg/ml) for 6- hours. The PBS plus alum inoculated mice were taken as control. The numbers in the contour plot represents the frequency of the population within the gate. Scatter plot represents the T-Bet+CXCR3+CD4+ T cells gated on CD4+ CD3+ T cells in all the 5 mice within the experiment. **(B)** Cytokine function of T_H1_ CD4+ T cell subset. Contour plots shows IFN-γ (top panel), TNF-α (middle panel) and IL-2 (bottom panel) secretion by T_FH_ CD4+ T cell subsets by flow cytometry in mice immunized with DSV4 and stimulated with EDIII1-4 peptide pool (10 μg/ml) for 6- hours. Mice immunized with PBS plus alum were taken as control. The numbers in the contour plot represents the frequency of the population within the gate. Scatter plot represents the IFN-γ+ (top), TNF-α+ (bottom) and IL-2+ (bottom) T_H1_ CD4+ T cell subsets gated T-Bet+CXCR3+CD4+ T cells gated in all the 5 mice within the experiment. **(C)** T_H1_ cytokine polyfunctionality. Bar graph shows the average frequencies of the indicated cytokine secreting population of the T_H1_ CD4+ T cell subset in mice immunized with DSV4 and stimulated with EDIII1-4 peptide pool (10 μg/ml) for 6- hours. The line in the scatter plot represents the geometric mean of the IFN-γ response in all the 5 mice. Each data point in the scatter plot represents an individual animal. The data shown is of a single experiment representative of two independent experiments with n=4-5 per group per experiment. **-p=0.001-0.01.

Intracellular pathogens and viruses primarily activate Type 1 helper (T_H1_) CD4+ T cells which are significant producers of inflammatory cytokines like IFN-γ and TNF-α. As shown previous experiment, we observed a higher IFN-γ response in DSV4 immunized mice and thus, here, we have looked at whether the viral dengue vaccine candidate, DSV4, induces the T_H1_ CD4+ T cells. In contrast to PBS plus alum injected control mice, DSV4 immunized mice had a higher frequency of T_H1_ CD4+ T cells on stimulation with dengue EDIII 1-4 peptide pool (**Figure 4A**). T_H1_ T cells were identified as CD4+ T cells expressing the transcription factor T-Bet and chemokine receptor CXCR3. These T_H1_ CD4+ T cells were also positive IFN-γ, TNF-α and IL-2 (**Figure 4B**), the typical cytokine secretion associated with T_H1_ CD4+ T cells. The IFN-γ-secreting T cells are a measure to assess the vaccine-induced immune responses. However, it is increasingly becoming evident that induction of polyfunctional T cells is essential and is a better correlate to determine long-term protective immune responses (20). Polyfunctional T cells exhibit multiple functions, like co-producing various cytokines. In this study, we looked at T_H1_ CD4+ polyfunctional T cells that can secrete two or more cytokines, IIFN-γ + TNF-α+, IFN-γ + IL-2+, TNF-α + IL2+ or IFN-γ + TNF-α + IL2+ (**Figure 4C**). Polyfunctional T_H1_ cells are superior in their cytolytic activity by secreting both IFN-γ and TNF-α, which are synergistic of each other. These T_H1_ CD4+ T cells also possess higher expansion and survival potential as they secrete IL-2, which enhances the cytolytic activity of T cells by increasing the expression of perforin and granzyme (20). On accessing the combination of cytokines secreted and co-producing T_H1_ CD4+ T cells, we observed that DSV4 immunization in mice results in few multifunctional T cells with the IFN-γ + TNF-α+ dual producers being highest and T cells secreting IFN-γ + TNF-α + IL-2+ being the lowest in frequencies. The T_H1_ response was dominated by single cytokine-secreting CD4+ T cells, particularly monofunctional TNF-α+ T_H1_ CD4+ T cells, followed by IFN-γ + and IL2+ T_H1_ CD4+ T cells. While we observed a higher T_H1_ CD4+ T cells, there was no significant difference in TH2 CD4+ T cell response (data not shown), both in frequencies (GATA3+CXCR4+CD4+) and cytokine functions (IL-4+ within GATA3+CXCR4+CD4+ T_H2_ gated cells) in EDIII 1-4 peptide pool stimulated DSV4 immunized mice. Thus, our data show that the dengue vaccine candidate DSV4 results in a T_H1_-dominated cellular immune profile and induction of polyfunctional co-producing T_H1_ CD4+ T cells in mice.

## Discussion

Dengue is a global epidemic resulting in over 100 million clinical cases each year (1), with symptoms ranging from mild self-limiting febrile illness to more severe dengue illness like dengue haemorrhagic fever (DHF) or dengue shock syndrome (DSS). This poses enormous challenges to health care systems (2). However, the search and need for a safe type-specific dengue vaccine is still ongoing. The tetravalent dengue subunit VLP-based vaccine candidate, DSV4, has shown in the mice (16). DSV4 eliminates ADE-competent epitopes in its construct and does not promote ADE of either DENV or ZIKA virus *in vivo* (10), providing significant protection against lethal dengue infection in mice. While most vaccines aim to develop a solid humoral and neutralizing antibody response against the pathogen, an effective vaccine candidate should be able to stimulate both the B-cell mediated humoral immunity, and T-cell mediated cellular immunity (21, 22). The focus of vaccinology is rapidly shifting to generate T cell responses along with neutralizing antibodies, which can mediate pathogen clearance and provide help to B cells leading to protective antibody responses. T cell responses help prevent viral replication and their propagation by killing virus-infected cells. Here in this study, we have shown that the dengue vaccine candidate, DSV4, induces robust dengue EDIII-specific T-cell response in mice, thus making it a promising vaccine candidate against dengue.

The protective role of T-cells during viral infection is well known, and dengue infection activates a broad range of T-cell responses against the virus in humans (23). Cytotoxic or CD8+ T cells mediate direct cytotoxicity and kill virus-infected cells. In contrast, helper or CD4+ T cells exhibit anti-viral mechanisms by promoting B cell and CD8+ T cell response, secreting anti-viral cytokines and mediating memory responses (24). Human anti-dengue T cell response is targeted majorly against non-structural proteins NS1, NS3, NS4b and NS5 by CD8+ T cells, while CD4+ T cells target structural proteins capsid, envelope, along with NS1, NS3, NS2 and NS5 dengue protein (18, 19, 25). Our dengue vaccine candidate, DSV4, comprises a part of the dengue envelope protein-EDIII region from all four serotypes fused with Hepatitis B surface antigen (HBsAg) for its self-assembly into a VLP (16). Our study shows that DSV4 immunization in mice results in secretion of IFN-γ by T-cells when they are re-stimulated with dengue EDIII 1-4 peptide pool (**Figure 1**). IFN-γ secretion is taken as a measure of active T-cell response induced by infection and vaccination. This assay uses a model of antigen presentation *in vitro* in the form of a dengue EDIII peptide pool that is presented on the MHC molecules of the antigen-presenting cells (APCs) to the antigen-specific T cells generated during DSV4 immunization. This leads to T cell activation and secretion of anti-viral cytokines, including IFN-γ, which is taken as a representative cytokine produced during viral infection or vaccination. This dengue EDIII-specific IFN-γ T cell response was seen equally among both CD4+ and CD8+ T cells, showing that dengue EDIII can serve as an epitope for both CD4+ and CD8+ T cells. This data is promising because although dengue EDIII is not an immunodominant T cell epitope, we observed antigen-specific IFN-γ response in DSV4 immunized mice that lasted more than a month (**Figure 2**). Dengue EDIII-specific T-cell response was also observed in a study where polyvalent dengue EDIII-NS1 DNA vaccine candidate was used to immunize mice (26). One limitation of our study is that we cannot say whether this dengue EDIII-specific T cell response is type-specific or cross-reactive. However, as EDIII is known to generate type-specific neutralizing antibodies, as reported previously (16), it might be possible that the T-cell response induced against EDIII in DSV4 immunization is also type-specific. This would then rule out the cross-reactive T cells that are generated against dengue during natural infection or with whole dengue virus-based vaccines. These cross-reactive T cells are ineffective, having low affinity and increasingly becoming known to result in pathological effects leading to severe dengue, just like the cross-reactive non-neutralizing antibodies. One of the future directions is to look at whether the dengue EDIII epitope can generate a cellular T cell response specific to different serotypes and not cross-reactive.

T follicular helper CD4+ T cells (T_FH_) play a crucial role in inducing germinal center (GC) reaction where T_FH_ cells and B cells interact. The B cells present antigen to T follicular helper cells (T_FH_ cells) *via* T cell receptor (TCR) and activate T cells (27). The activated T cells then provide proliferating and cytokine signals to B cells. T_FH_ cells secrete IL-21 and IL-10, allowing proliferation and differentiation into GC B effector cells. They also lead to the generation of high affinity matured antibodies that display higher and better neutralizing antibodies resulting in an overall high-quality humoral response. T_FH_ cells have been shown to be protective in humans during viral infections and vaccinees. It has also been previously studied that T_FH_ cells expand during dengue infection and promote dengue-specific antibody response in mice (28). Our study shows that immunization with DSV4 in mice, an expansion of EDIII-specific T_FH_ cells is observed that are activated and proliferating and possess the ability to secrete IL-21 and IL-10 (**Figure 3**). Previous studies have shown that T_FH_ CD4+ T cells promote B cell responses and expand the GC B cells, effector B cells called plasmablasts and neutralizing antibodies secreted. TFH cells is also believed to boost memory response by generating long-lived plasma cells and memory B cells of high affinity. It has also been studied that in mice with during homologous dengue infection and flavivirus cross-reactive infection, T_FH_ cells lead to higher avidity antibodies that have higher neutralizing capacity (29). However, our study lacks how these activated T_FH_ cell frequencies and their function correlate with the humoral response generated in DSV4 immunized mice and is needed to be investigated in detail.

During a viral infection and vaccination, a T_H1_-dominated T cell response is observed in both mice and humans. These T_H1_ cells mediate viral clearance and secrete anti-viral inflammatory cytokines like IFN-γ and TNF-α (30). Previous studies have shown that dengue infection induces T_H1_ immune responses in humans, with a serum cytokine profile showing T_H1_ cytokines in the live attenuated dengue vaccine recipients (18, 31). The EDIII-NS1 DNA vaccine also elicited TH1-predominant responses with higher IgG2/IgG1 ratio in mice (32). Indeed, our study also demonstrated that DSV4 immunization in mice resulted in T cells acquiring T_H1_ phenotype with expression of CXCR3 and T-Bet. The induced T_H1_ CD4+ T cells also secreted substantial amounts of T_H1_ cytokines IFN-γ, TNF-α and IL-2 (**Figure 4**).

The induction of polyfunctional T cells is considered an essential element of T cell immune response following vaccination. Importantly, people worldwide are becoming increasingly aware that accessing the magnitude of IFN-γ response may not serve as a sufficient correlate of protection conferred by T-cells after vaccination. IFN-γ mono producers T cells comprise a significant fraction of activated T cells following immunization; however, they have limited capacity to be sustained as memory T cells (33). Thus, apart from looking at the “magnitude” of IFN-γ-producing T cells, which governs the number of responsive T cells, it is also essential to understand the “quality” of the T cell response, which is dictated by the nature and diversity of the functional response. Better immune response in terms of quality can lead to protective memory T cell response. Polyfunctional T cells secreting multiple cytokines have been shown to contain microbial and viral infections in humans. Polyfunctional T_H1_ T cells are functionally superior to monofunctional IFN-γ-secreting T cells (34). These polyfunctional T cells secrete more IFN-γ on a per cell basis than monofunctional IFN-γ producers and elicit stronger cytolytic activities as they secrete both IFN-γ and TNF-α which complement each other actions. IL-2 secretion mediates proliferation, maintenance, long term survival of T cells and also provides help to CD8+ T cells. Here, we have reported that immunization with DSV4 results in the induction of polyfunctional T_H1_ CD4+ T cells that can secrete one, two or all three cytokines, namely IFN-γ, TNF-α and IL-2. However, further studies are needed to assess the role of these polyfunctional T cells in the mice’s viral clearance and their progression to severe disease.

Sanofi Pasteur’s Dengvaxia (CYD-TDV), a vaccine for dengue, protects only dengue seropositive individuals while priming dengue naïve individuals for the severe disease once infected post-vaccination (35). Another dengue vaccine candidate, Takeda (TAK-003), which uses a live attenuated strain of DENV-2 with PrM and E of different serotypes, elicits a robust humoral response along with the generation of polyfunctional CD8+ T cell response (15), which is serotype cross-reactive. However, whether or not this vaccine enhances infection *via* ADE is still controversial. Our dengue vaccine candidate, DSV4, a virus-like particle comprised of the EDIII region of all four serotypes with HBsAg, showed high neutralizing antibody titers in mice against all four DENV serotypes, which is type-specific and does not cross-react (16). It has also been shown that in experimental ADE mice models, DSV4 minimizes the risk of ADE and do not result in enhancement of either DENV or ZIKV infection *in vivo* (10). Here we have dissected the T cell response, which is induced by DSV4 immunization in mice. Our results indicate that the tetravalent VLP dengue vaccine candidate, DSV4, can serve as an effective dengue vaccine. Apart from inducing type-specific dengue neutralizing antibodies, it also elicits a robust cellular immune T-cell response.

Our data show that DSV4 immunization in mice leads to the development of not only IFN-γ-producing T cells but also dengue EDIII-specific T_FH_ CD4+ T cells and polyfunctional T_H1_ cells. We have dissected deeper into CD4+ T cell response since CD4+ T cells are the major players that help B cells and CD8+ T cells and contribute to memory responses. The CD8+ T cell response needs to be studied further, and its role in protection needs to be elucidated.

In summary, our study shows that the immunization of mice with DSV4 results in the induction of T-cell response that is governed by IFN-γ-secreting T cells post re-stimulation with dengue EDIII1-4 peptide pool. It also results in a marked increase in T_FH_ CD4+ T cell response and T_H1_ CD4+ T cells that are also polyfunctional, indicating that DSV4 immunization leads to both higher magnitude and quality of T cell response in mice. Our data suggest that DSV4 can serve as an effective dengue vaccine candidate resulting in activation of both arms of immunity- humoral and cellular immunity.

### Statistical analyses

GraphPad prism software (version 9.3.) was used for all the statistical calculations and Mann-Whitney test was used to determine statistical significance of the difference between data sets. Probability (p) value, *p<0.05* was considered as significant and p<0.0001 observed as very significant.

## Data availability statement

The original contributions presented in the study are included in the article/**Supplementary Material**. Further inquiries can be directed to the corresponding authors.

## Ethics statement

All the experiments using BALB/c mice were conducted at International Centre for Genetic Engineering and Biotechnology (ICGEB), New Delhi (IAEC No.: ICGEB/IAEC/07032020), in compliance with the guidelines of the Committee for the Purpose of Control and Supervision of Experiments on Animals (CPCSEA) of the Government of India.

## Author contributions

Literature search: CA, RS, and NK; DSV4 antigen preparation: VR; DSV4 formulation and immunization: RS and AG, figures: CA, RS, AG, and NK; study design: CA, RS, and NK; data collection: CA, AG, and RS; data analysis and interpretation: CA, RS, AG, and NK; writing first draft: CA, RS, and NK; final editing: CA, RS, and NK. All authors contributed to the article and approved the submitted version.
