# Supplementary material for: Cellular T-cell immune response profiling by tetravalent dengue subunit vaccine (DSV4) candidate in mice
Source: Front Immunol. 2023 Feb 28;14:1128784. doi: 10.3389/fimmu.2023.1128784 (PMC10011089; doi:10.3389/fimmu.2023.1128784)
Supplement: Supplementary file 1 [file DataSheet_1.pdf]

Supplementary table 1: Custom synthesized peptide library of all four EDIII (EDIII-1, -2, -3 and -4) of DENV.

DENV-EDIII (~104 aa)[#]	Peptide code	Peptide sequence	Peptide length* (aa)	Intermediate Peptide pool	Final peptide pool
DENV-1-EDIII (EDIII-1)	EDIII-1_1	MSYVMCTGSF	10	Pool of EDIII-1 peptides (0.1 mg each peptide)	EDIII-1, 2, 3 and -4 peptides pool (0.5 mg each EDIII peptides pool) **Working concentration: 10 µg/ml**
	EDIII-1_2	GSFKLEKEVA	10		
	EDIII-1_3	EVAETQHGTV	10		
	EDIII-1_4	GTVLVQVKYE	10		
	EDIII-1_5	KYEGTDAPCK	10		
	EDIII-1_6	PCKIPFSSQD	10		
	EDIII-1_7	SQDEKGVTQN	10		
	EDIII-1_8	TQNGRLITAN	10		
	EDIII-1_9	TANPIVTDKE	10		
	EDIII-1_10	DKEKPVNIEA	10		
	EDIII-1_11	IEAEPPFGES	10		
	EDIII-1_12	GESYIVVGAG	10		
	EDIII-1_13	GAGEKALKLS	10		
	EDIII-1_14	KLSWFKKGSS	10		
	EDIII-1_15	GSSIGK	6		
DENV-2-EDIII (EDIII-2)	EDIII-2_1	MSYSMCTGKF	10	Pool of EDIII-2 peptides (0.1 mg each peptide)	
	EDIII-2_2	GKFKVVKEIA	10		
	EDIII-2_3	EIAETQHGTI	10		
	EDIII-2_4	GTIVIRVQYE	10		
	EDIII-2_5	QYEGDGSPCK	10		
	EDIII-2_6	PCKTPFEIMD	10		
	EDIII-2_7	IMDLEKRHVL	10		
	EDIII-2_8	HVLGRLTTVN	10		
	EDIII-2_9	TVNPIVTEKD	10		
	EDIII-2_10	EKDSPVNIEA	10		
	EDIII-2_11	IEAEPPFGDS	10		
	EDIII-2_12	GDSYIIIGVE	10		
	EDIII-2_13	GVEPGQLKLD	10		
	EDIII-2_14	KLDWFKKGSS	10		
	EDIII-2_15	GSSIGQ	6		
DENV-3-EDIII (EDIII-3)	EDIII-3_1	MSYAMCLNTF	10	Pool of EDIII-3 peptides (0.1 mg each peptide)	
	EDIII-3_2	NTFVLKKEVS	10		
	EDIII-3_3	EVSETQHGTI	10		
	EDIII-3_4	GTILIKVEYK	10		
	EDIII-3_5	EYKGEDAPCK	10		
	EDIII-3_6	PCKIPFSTED	10		
	EDIII-3_7	TEDGQGKAHN	10		
	EDIII-3_8	AHNGRLITAN	10		
	EDIII-3_9	TANPVVTKKE	10		
	EDIII-3_10	KKEEPVNIEA	10		
	EDIII-3_11	IEAEPPFGES	10		
	EDIII-3_12	GESNIVIGIG	10		
	EDIII-3_13	GIGDKALKIN	10		
	EDIII-3_14	KINWYRKGSS	10		
	EDIII-3_15	GSSIGK	6		
DENV-4-EDIII (EDIII-4)	EDIII-4_1	MSYTMCSGKF	10	Pool of EDIII-4 peptides	
	EDIII-4_2	GKFSIDKEMA	10		
	EDIII-4_3	EMAETQHGTT	10		

	EDIII-4_4	GTTVVKVKYE	10	(0.1 mg each peptide)	
	EDIII-4_5	KYEGAGAPCK	10		
	EDIII-4_6	PCKVPIEIRD	10		
	EDIII-4_7	IRDVNKEKVV	10		
	EDIII-4_8	KVVGRIISPT	10		
	EDIII-4_9	SPTPFAENTN	10		
	EDIII-4_10	NTNSVTNIEL	10		
	EDIII-4_11	IELERPLDSY	10		
	EDIII-4_12	DSYIVIGVGD	10		
	EDIII-4_13	VGDSALTLHW	10		
	EDIII-4_14	LHWFRKGSSI	10		
	EDIII-4_15	SSIGK	5		

#Peptide library was custom synthesised from GenScript, USA with the purity of ≥90% for each peptide. The quality and purity of peptide were identified through RP-HPLC, and MS for each peptide and lyophilized peptides were received. Each EDIII-1, -2, -3 and -4 encodes from ~104 amino acids (aa) corresponding to WHO referenced DENV-1 West Pac-74, DENV-2 PR159-S1/69, DENV-3 H87/56 and DENV-4 H241-P, respectively.
**The length of peptides (1-14) were 10 aa with the 3- aa overlap for each EDIIIs (EDIII-1, -2, -3 & -4) and the length of last peptide of EDIII-1-3 was 6-aa and for EDIII-4 was 5-aa as EDIII-4 having total 103 aa.*

Supplementary table 2: Custom synthesized HBsAg peptide library

Peptide#	Peptide code	Peptide sequence	Peptide length (aa)	HBsAg peptide pool
HBsAg peptide (226 aa)	Peptide_1	MENITSGFLG	10	HBsAg peptide pool (0.1 mg of each peptide of 1-32)<br><br>**Working concentration: 10 µg/ml**
	Peptide_2	FLGPLLVLQA	10	
	Peptide_3	LQAGFFLLTR	10	
	Peptide_4	LTRILTIPQS	10	
	Peptide_5	PQSLDSWWTS	10	
	Peptide_6	WTSLNFLGGS	10	
	Peptide_7	GGSPVCLGQN	10	
	Peptide_8	GQNSQSPTSN	10	
	Peptide_9	TSNHSPTSCP	10	
	Peptide_10	SCPPICPGYR	10	
	Peptide_11	GYRWMCLRRF	10	
	Peptide_12	RRFIIFLFIL	10	
	Peptide_13	FILLLCLIFL	10	
	Peptide_14	IFLLVLLDYQ	10	
	Peptide_15	DYQGMLPVCP	10	
	Peptide_16	VCPLIPGSTT	10	
	Peptide_17	STTTSTGPCK	10	
	Peptide_18	PCKTCTTPAQ	10	
	Peptide_19	PAQGNSMFPS	10	
	Peptide_20	FPSCCCTKPT	10	
	Peptide_21	KPTDGNCTCI	10	
	Peptide_22	TCIPIPSSWA	10	
	Peptide_23	SWAFAKYLWE	10	
	Peptide_24	LWEWASVRFS	10	
	Peptide_25	RFSWLSLLVP	10	
	Peptide_26	LVPFVQWFVG	10	
	Peptide_27	FVGLSPTVWL	10	
	Peptide_28	VWLSAIWMMW	10	

	Peptide_29	MMWYWGPSLY	10	
	Peptide_30	SLYSIVSPFI	10	
	Peptide_31	PFIPLLPIFF	10	
	Peptide_32	IFFCLWVYI	9	

#HBsAg amino acid (aa) sequence was obtained from HBV adw serotype and HBsAg peptide library (10 aa long peptide with 3 aa overlap) was custom synthesised from GenScript, USA.
